# Comparative Analysis of the ABC/2 Score and e-ASPECTS Software in the Determination of Acute Ischaemic Stroke Volume from Non-Contrast CT

**DOI:** 10.3390/brainsci15060560

**Published:** 2025-05-24

**Authors:** Jorin Bejleri, Sarah Power, Fiona Boland, Olivier Joly, David J. Williams, John J. Thornton, Shona Pfeiffer

**Affiliations:** 1 Department Physiology & Medical Physics, RCSI University of Medicine and Health Sciences, D02 YN77 Dublin, Ireland; jorinbejleri@gmail.com; 2 Department Geriatric & Stroke Medicine, Beaumont Hospital, RCSI University of Medicine and Health Sciences, D09 YD60 Dublin, Ireland; 3 Department Neuroradiology, Beaumont Hospital, D09 V2N0 Dublin, Ireland; sarahpower@beaumont.ie (S.P.); johnthornton@beaumont.ie (J.J.T.); 4 Data Science Centre, RCSI University of Medicine and Health Sciences, D02 YN77 Dublin, Ireland; fionaboland@rcsi.ie; 5 Brainomix Ltd., Oxford OX2 0JJ, UK; ojoly@brainomix.com

**Keywords:** ABC/2, ischaemic stroke, infarct measurement, infarct volume, non-contrast computerised tomography, stroke

## Abstract

Background and Purpose: Accurate and reproducible methods for assessing infarct volume in acute ischaemic stroke have important therapeutic and prognostic implications for the choice and success of acute therapeutic interventions. However, there is no international consensus on the methodology employed in infarct volume assessment. We aimed to assess the reliability of the ABC/2 score and e-ASPECTS in the determination of infarct volume in acute ischaemic stroke. Methods: Infarct volume was measured from NCCT in stroke patients recruited ≤12 h of symptoms onset and at 24 h using the ABC/2 method. Automated ischaemic volume measurements were carried out using e-ASPECTS software. Measurements using ABC/2 were compared with e-ASPECTS to assess volume differences and reliability using Lin’s concordance correlation coefficient. Results: Thirty-three patients with CT < 12 h from onset of symptoms and follow-up at 24 h were included in the analysis. Use of ABC/2 demonstrated low agreement between observers (0.490, CI 0.236–0.743, *p* < 0.001) on admission (<12 h). High agreement was found between observers at 24 h (0.724, CI 0.564–0.884, *p* < 0.001). High agreement was observed between the mean observed infarct volumes using ABC/2 and e-ASPECTS on admission (0.794, CI 0.691–0.898, *p* < 0.001). Conclusions: Our results suggest that e-ASPECTS is a reliable platform for ischaemic volume determination particularly in the hyperacute phase to inform management. However, the use of ABC/2 represents an alternative approach to e-ASPECTS in the rapid and reliable estimation of ischaemic infarct volume to inform prognosis and treatment decisions, particularly in cases of delayed presentation where infarction is established and arterial territory boundaries are easily identifiable.

## 1. Introduction

The accurate and reliable determination of infarct volume is integral to a patient-centred care approach as the treatment of ischaemic stroke is time-dependent [1]. Considerable clinical research has been carried out to establish methods for the assessment of ischaemic stroke lesions using non-contrast computerised tomography (NCCT) and magnetic resonance imaging (MRI) to guide acute stroke treatment. Ischaemic volume is a valuable tool to improve patient selection for treatment and measure its efficacy [2,3]. New state-of-the-art non-invasive CT perfusion imaging modalities have been successfully employed not only to select patients for large-scale stroke clinical trials [4,5] but also to select those who would benefit from reperfusion therapy based on an accurate assessment of the salvageable tissue at risk of infarction without intervention [6]. Evidence suggests that infarct volume determination is a reliable prognostic tool for functional outcome, and infarct volume assessed by perfusion imaging can be used as a marker to guide treatment in patients with unknown time of onset of stroke symptoms [6,7]. Moreover, there is now accumulating evidence which suggests that patients with large lesions on diffusion-weighted magnetic resonance imaging (DWI-MRI) have a high risk of developing symptomatic intracerebral haemorrhage (ICH), leading to a worse clinical outcome following intravenous thrombolysis [8]. Therefore, accurate measurement of the ischaemic volume in the acute phase represents a robust radiological marker for treatment guidance, the prediction of treatment adverse effects, and for the evaluation of the success of therapeutic intervention.

Despite progress being made in the validation of infarct volume using imaging techniques such as MRI and perfusion imaging, there are major limiting factors that hinder the wider use of infarct volume, as assessed by these diagnostic imaging techniques in the clinical setting. This is primarily attributable to the limited access to MRI, in particular in remote non-tertiary centres, which remains challenging, and the time delay introduced when performing MRI represents a major concern in the setting of acute ischaemic stroke. In addition, the interpretation of MRI is operator-dependent and requires a highly specialised and skilled neuroradiologist for rapid and accurate evaluation. Baseline CT and CT angiogram are the primary and most accessible tools used for patients with suspected acute stroke in most tertiary stroke centres. Maximisation of the radiological information from CT scanning, therefore, such as the assessment of acute stroke volume, together with clinical expertise, represents the cornerstone of the initial management of acute stroke patients.

To date, there is no international consensus on a reliable and reproducible methodology for the determination of stroke infarct volume in the acute phase. ABC/2 is a simple and rapid manual method employed in the assessment of ICH volume [9]. There is now growing evidence supporting the use of ABC/2 as a reliable method in the measurement of infarct volume [10,11]. Moreover, meaningful progress has been made in the development of automated software in the evaluation of NCCTs through the e-ASPECTS software (Brainomix, UK), an ASPECTS scoring tool which offers the quantification of automatically derived acute ischaemic volume (AAIV) of the anterior cerebral circulation territory [12]. We performed an exploratory comparative analysis of the acute stroke volume measurement, as assessed by ABC/2, and automatically derived acute ischaemic volumes, as assessed by e-ASPECTS software.

## 2. Subjects and Methods

### 2.1. Patients

Measurements using the ABC/2 method were performed independently by two experienced diagnostic and interventional neuroradiologists who were not blinded to the clinical information on 33 patients recruited to the miRNA as the Novel Diagnostic biomarkers (MiND) study from July 2019 to November 2021. Neuroradiologists were blinded to the results of the measurements calculated by e-ASPECTS. The MiND study is an Irish-led study assessing the diagnostic and prognostic biomarkers of acute ischaemic stroke, recruiting acute stroke patients within 12 h from symptoms onset. Patients recruited to MiND were included in this analysis if they were ≥18 years of age, neuroimaging was consistent with acute ischaemic stroke, and ≤12 h from symptom onset. We included all consecutive patients who at the time of the study had their initial NCCT ≤ 12 h of symptom onset and follow-up scan at 24 h. Thirty-three cases meeting the inclusion criteria who had follow-up CT 24 h after admission and had anterior circulation territory stroke were selected for further analysis. We excluded cases who had posterior circulation and lacunar infarcts from this analysis as e-ASPECTS is currently intended for large vessel occlusion stroke (LVO). For each patient, demographic, clinical, and radiological data were retrospectively collected (Supplementary Table S1). Neuroradiologists were blinded to the results of the measurements calculated by e-ASPECTS. This study is reported following the Standards for Reporting Diagnostic Accuracy Study (STARD) guidelines. This study was approved by Institutional Research Ethics Committees. Informed written consent was obtained from all patients.

### 2.2. Measurements

**Figure 1:**
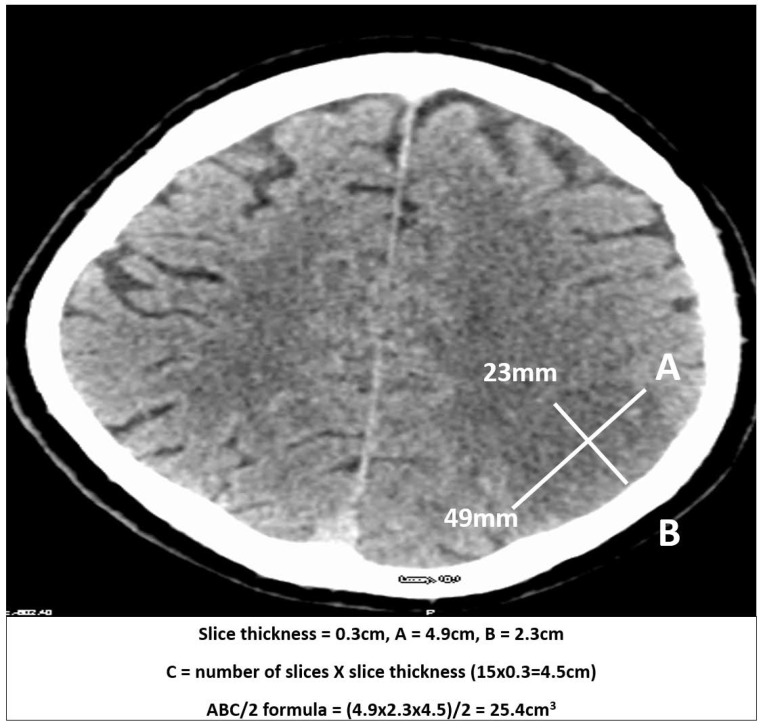
Infarct volume assessment using the ABC/2 formula. A is the largest diameter of the selected slice; B is the largest diameter perpendicular to A.

Acute ischaemic volume was measured from NCCT performed on admission (≤12 h) and at 24 h (*n =* 33). Slice thickness of CT ranged from 2.5 to 5 mm. Infarct volume (cm^3^) was measured using the ABC/2 formula, calculated as A*B*C/2, where (A) is the largest diameter of the selected slice, (B) is the largest diameter perpendicular to (A), and (C) is the total number of slices of infarct multiplied by slice thickness (C) (Figure 1). In cases where the infarct appeared to be a combination of several discrete lesions, the volume was determined by calculating an average volume from all the infarct lesions identified. For ease of comparison, ischaemic stroke volume has been converted to ml (1 cm^3^ = 1 mL).

### 2.3. Automated Ischaemic Volume by e-ASPECTS

Automated ischaemic volume measurements were carried out on NCCT performed on admission using e-ASPECTS software (Brainomix, Oxford, UK). The e-ASPECTS image-processing algorithm assesses for acute ischaemic changes and non-acute hypodensities, and volumetric values are given for both left and the right hemispheres. The acute map highlights the voxels in the image that are typical of early ischaemic changes, and the sum of the voxels is converted into a volumetric value in millilitres. AAIV was calculated in all of the scored ASPECTS regions of the hemisphere involved in the acute stroke.

### 2.4. Statistical Analysis

Tests for normality and equal variance, scatter plots, and paired *t*-tests were conducted to explore potential differences between individual observer measurements and between ABC/2 and e-ASPECTS. Linear regression and Bland–Altman plots were used to explore the relationship between the mean observed infarct volume and e-ASPECTS. Lin’s concordance correlation coefficient was used to identify the inter-rater reliability of ABC/2 and AAIV by e-ASPECTS. *p* < 0.05 was considered to be statistically significant. All analyses were performed using Stata/SE V16.0.

## 3. Results

### 3.1. Clinical Characteristics

Thirty-three patients with CT < 12 h from the onset of symptoms and follow-up at 24 h were included in the analysis. The baseline characteristics are presented in Table 1. The mean age of the acute ischaemic patients was 75 years (SD ± 10.8), and 42% of the cohort were female. The most common risk factors for stroke reported were hypertension (67%) and dyslipidaemia (64%), followed by ischaemic heart disease (39%). Stroke aetiology was classified based on TOAST (Trial of ORG 10172 in Acute Stroke Treatment) criteria, and 52% of strokes have cardioembolic (Cemb) aetiology, while 30% of the cohort had cryptogenic stroke. A total of 58% of the cohort had the M1 segment of the middle cerebral artery (MCA) identified as the occlusion site, with 3% having no occlusion site identified. Most patients were treated with either intravenous tissue plasminogen activator (IV tPA) (70%) or endovascular thrombectomy (EVT) (70%) or both. The high number of patients receiving reperfusion therapy relates stringent inclusion criteria that were employed for the MiND study where patients were recruited only if they were within 12 h of symptoms onset or last seen well (LSW), making them eligible for treatment.

### 3.2. Infarct Volume

**Figure 2:**
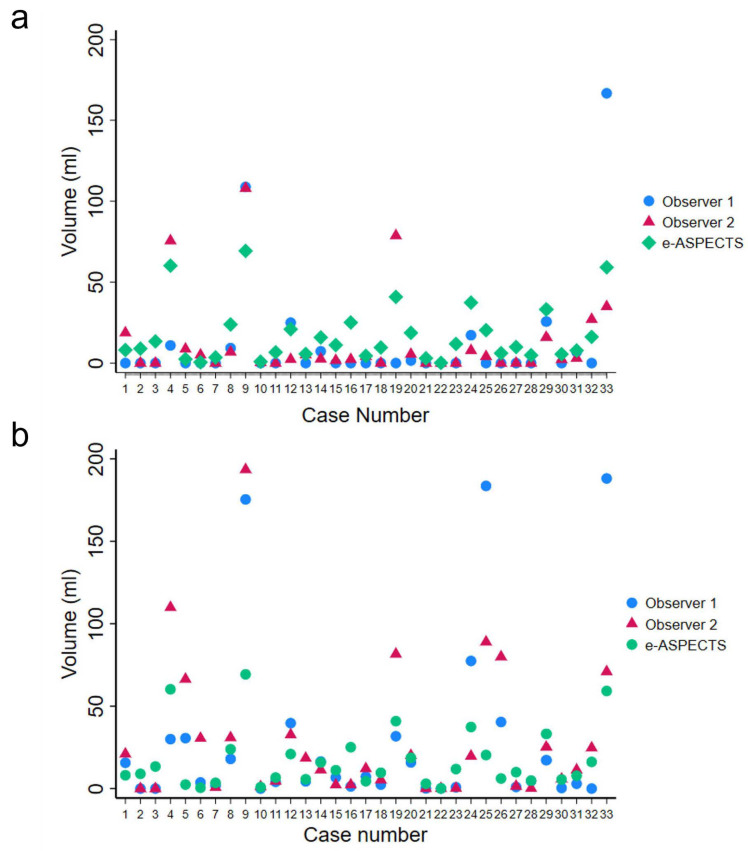
Acute infarct volume measurements for each individual case as measured from NCCT by observers 1 and 2 using the ABC/2 method, and by e-ASPECTS (**a**) on admission and (**b**) at 24 h (*n* = 33).

Infarct volumes measured for each individual case from NCCT using the ABC/2 formula both on admission and at 24 h, and e-ASPECTS on admission, are shown in Figure 2. e-ASPECTS is developed to measure the acute infarct volume rather than the chronic infarct; therefore, we measured the acute infarct volume using the e-ASPECTS software only on CTs performed on admission because the infarct in the follow-up scans becomes more established and less acute, leading to underestimation of the infarct volume by the software. We compared the infarct volume measured by e-ASPECTS on admission with the infarct volume derived from the ABC/2 formula for CT performed on admission and at 24 h to assess for infarct volume difference between both methods.

Infarct volume measurements among observers using ABC/2 on admission ranged from 0 to 166.7 mL (observer 1), 0 to 108 mL (observer 2), and 0 to 69.3 mL for e-ASPECTS (Figure 2a) and from 0 to 188.1 mL (observer 1) and 0 to 193.5 mL (observer 2) at 24 h (Figure 2b).

Using ABC/2, there was no difference between both observer measurements for 33 cases on admission (−1.3 mL, confidence interval (CI) −12.1–9.5, *p* = 0.81) and at 24 h (−1.6 mL, CI −14.5–44.5, *p* = 0.80) (Table 1). There was a significant difference between the mean observer volume on admission but not at 24 h compared to mean infarct volume as measured by e-ASPECTS software (−5.0 mL, CI −9.8–−0.2, *p* = 0.04 on admission and 11.6 mL, CI −0.1–23.2, *p* = 0.05 at 24 h) (Table 1).

### 3.3. Interobserver Agreement

Interobserver agreement was measured using Lin’s concordance correlation coefficient (Table 2). There is a low agreement between observer 1 and 2 (0.490, CI 0.236–0.743, *p* < 0.001) for CTs on admission using ABC/2. In follow-up CT scans performed at 24 h, a high degree of agreement was found between both observers (0.724, CI 0.564–0.884, *p* < 0.001).

High agreement was observed between the mean of both observers’ infarct volumes and e-ASPECTS on admission (0.794, CI 0.691–0.898, *p* < 0.001). When analysing the level of agreement of individual observers with e-ASPECTS, observer 2 had a high agreement with e-ASPECTS for CTs on admission (0.756, CI 0.629–0.883, *p* < 0.001), with a lower level of agreement for CTs at 24 h (0.492, CI 0.339–0.645, *p* < 0.001). A similar moderate level of agreement was seen between observer 1 and e-ASPECTS software on admission (0.576, CI 0.409–0.744, *p* < 0.001) and at 24 h (0.399, CI 0.252–0.546, *p* < 0.001).

**Figure 3:**
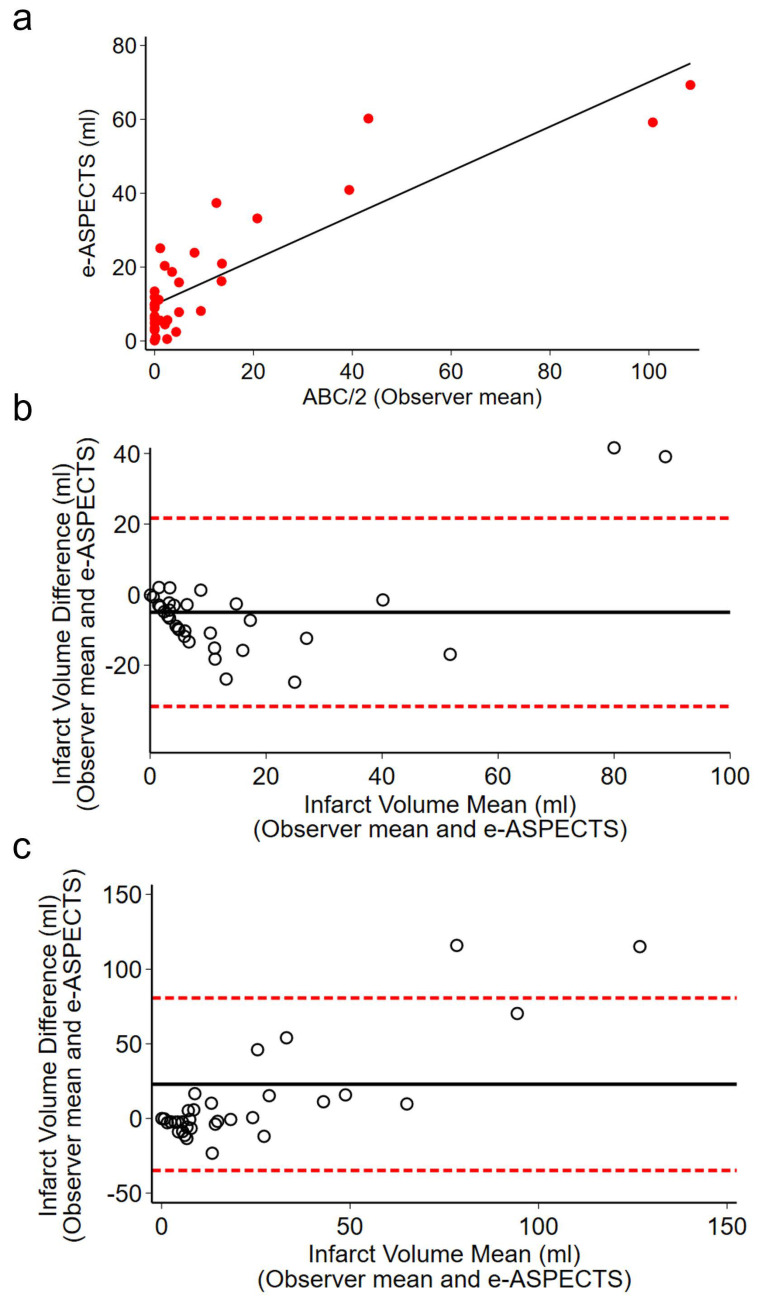
(**a**) Linear regression showing a positive relationship between manual ABC/2 (observer mean) and automated e-ASPECTS measurements on admission. (**b**,**c**) Bland–Altman plots comparing acute ischaemic infarct volume (ml) on admission (**b**) and 24 h (**c**) as measured by ABC/2 and e-ASPECTS (*n* = 33). Solid black line represents the mean difference between two methods; dashed lines indicate the limits of agreement (±1.96 SD from the mean difference).

Linear regression analysis of the relationship between ABC/2 and e-ASPECTS demonstrated a moderate relationship between the observers’ mean and e-ASPECTS (slope, [CI]; 0.6 [0.478–0.727], *p* < 0.001) on admission; however, this was not observed at infarct volumes <60 mL (Figure 3a). Bland–Altman plots demonstrated better agreement between the mean of observer 1 and 2 with e-ASPECTS on admission (mean difference −5.02, (−31.71–21.67)) compared to 24 h (mean difference 22.93, (−34.86–80.71)) (Figure 3b,c).

### 3.4. Sensitivity Analysis

We performed further sensitivity analysis in patients with confirmed LVOs (Supplementary Tables S2 and S3). No difference was seen between observer measurements on admission (−1.3 mL, CI −12.9–10.2, *p* = 0.81) and at 24 h (−1.7 mL, CI −15.1–11.7, *p* = 0.8). There is a low level of agreement between both observers on admission (0.484, CI 0.220–0.748, *p* < 0.001) but a high agreement at 24 h (0.717, CI 0.549–0.885, *p* < 0.001). Similarly, high agreement between the observers’ mean and e-ASPECTS is observed on admission (0.794, CI 0.687–0.900, *p* < 0.001) but not at 24 h (0.487, CI 0.335– 0.638, *p* < 0.001). Further sensitivity analysis performed by excluding outliers revealed no major difference in the data.

## 4. Discussion

We included in our study a variety of vessel occlusion sites and a range of infarct volumes, making the identification of the infarct boundary challenging and therefore more strongly dependent on the interpretation of a neuroradiologist. We used neuroimages from patients recruited to one tertiary centre, rendering the variability of CT scans’ quality, density scales, calibration, and slice thickness less likely. Apart from operator experience, the identification of boundaries in the acute phase of stroke also depends on the stroke site, and it has been previously demonstrated that interobserver agreement improves when structures are well demarcated [13]. Our initial analysis showed a low level of interobserver agreement using ABC/2 in the acute phase; this improved in volumes measured at 24 h as infarct becomes more established and infarct boundaries become more demarcated and more easily identifiable from CT. Similarly, when performing a sensitivity analysis of the cases with LVO, we re-demonstrated that in the acute phase, ABC/2 performs moderate–low, as reflected by the low agreement between observers. The initial infarct volumes, as measured by the observers, from CTs on admission reveal a smaller volume when compared with the volumes measured from CTs at 24 h. This could potentially be a result of infarct volume not being clearly visualised from the admission CT as it is at 24 h, leading to underestimation of the ‘real’ infarct volume. Alternatively, the infarct volumes, as measured by the observers, on admission are indicative of the infarct at the particular time when the CT was performed, and with ongoing ischaemia, the infarct evolves and results in the final infarct volume seen at 24 h.

Several studies have previously reported on the reproducibility and reliability of the ABC/2 method in the assessment of ICH and infarct volume from DWI and CT [9,11,14,15]. Despite the difficulty of defining infarct margins in the acute setting, particularly <3 h, we observed a low-to-high level of agreement when using ABC/2 among observers and between observers and e-ASPECTS on admission. The low level of agreement between observers on admission improved to high agreement when using ABC/2 in CTs at 24 h. This supports the use of ABC/2, particularly in delayed presentation. There is growing evidence supporting the achievement of a good functional outcome by EVT in patients with late presentation and established core infarct of 101–130 mL on DWI [16], and DWI infarct measurement using ABC/2 has been shown to be a reliable alternative to automated software in unknown/late-presentation strokes [17]. Our data suggest that the employment of ABC/2 using easily accessible and time-efficient imaging such as NCCT in late-presentation stroke (>12 h from symptoms onset or last seen well), which would also require CTA, represents an alternative simple, rapid technique in screening patients for reperfusion therapy. Unlike e-ASPECTS, ABC/2 can be applied in both anterior and posterior circulation stroke, while e-ASPECTS is validated in anterior circulation stroke only.

Considerable progress has been made in the development of automated software for the quantification of ischaemic volume in the acute phase [12]. We report a moderate–high level of agreement between observers, individually and in combination, and the e-ASPECTs score on admission. Furthermore, in our sensitivity analysis of cases with LVO, we re-demonstrated a high level of agreement between observers on admission CT, individually, and in combination with e-ASPECTS. e-ASPECTS is designed to assess infarct volume in the acute setting and is not designed for the ‘non-acute infarct’ as the infarct becomes more established at 24 h. Therefore, we did not use e-ASPECTS in the infarct volume estimation at 24 h as this would lead to under-segmentation of the infarct volume in follow-up scans. Indeed, the utility of e-ASPECTS has been previously demonstrated by the strong correlation of AAIV from NCCT with both CT perfusion and DWI-derived ischaemic core volumes in patients, with a mean symptom to imaging time <2.5 h, highlighting a very good sensitivity of e-ASPECTS in detecting early ischaemia in the hyperacute phase [12]. Furthermore, the integration of e-ASPECTS into stroke assessment aids decision-making regarding treatment options and triage of the patient to the most appropriate target hospital [18]. Accumulating evidence supports the utility of e-ASPECTS in different settings and patient populations, and it has been shown that e-ASPECTS is non-inferior to expert neuroradiologists [19]. In addition, e-ASPECTS scores are independent predictors of outcome and symptomatic ICH in patients undergoing mechanical thrombectomy or intravenous thrombolysis [20,21].

Our study supports this. We suggest that the use of a validated automated software such as e-ASPECTS can be viewed as a reliable and suitable tool in assessing stroke volume in the acute phase in the setting of a requirement for fast decision-making, where areas of hypointensity are not sharply demarcated, are difficult to assess manually, and are dependent on neuroradiology expertise. However, we observed underestimation of infarct size by e-ASPECTS when compared to observer volumes. Larger infarcts on follow-up imaging may be a result of ongoing infarction or progression in tissue already infarcted but not visualised in initial imaging. This will need further evaluation.

Our study is limited by the small sample size, and this cohort included a range of clinical and radiological characteristics, affecting the consistency of our results. It should also be noted that our findings only relate to one of several commercially available software tools; the use of other commercially available software could lead to different results, and these would need to be validated separately. Future prospective cohort studies with larger patient cohorts are warranted for the validation of our results.

## 5. Conclusions

Considerable heterogeneity in reliable and reproducible methodology employed to measure acute stroke infarct volume exists, and the choice of the most appropriate approach is strongly influenced by factors such as resource-limited settings or delayed presentation. This comparative analysis provides novel insights into the strengths and reliability in the use of the ABC/2 score and automatically derived acute ischaemic volumes, as assessed by e-ASPECTS software, with important implications for selection in different clinical contexts. Our results suggest that e-ASPECTS is reliable in the estimation of ischaemic volume in the hyperacute phase, particularly in smaller centres when expert neuroradiology is not available, better informing treatment management. However, the use of the ABC/2 formula, particularly in the delayed presentation scenario where infarct boundaries are more demarcated and where access to automated software is not available, is also a reliable technique, perhaps excluding the need for more complex imaging. Further evaluation is required.

## Figures and Tables

**Table 1:** Infarct volume as measured from CT on admission and at 24 h. O1, observer 1; O2, observer 2.

	O1	O2	Mean (O1, O2)	e-ASPECTS	O1 vs. O2		O1 vs. e-ASPECTS		O2 vs. e-ASPECTS		Mean (O1, O2) vs. e-ASPECTS	
	Volume (mL)				Diff (mL)	*p*	Diff (mL)	*p*	Diff (mL)	*p*	Diff (mL)	*p*
CT on admission (*n* = 33)	11.5 ± 34.1	12.8 ± 25.9	12.1 ± 26.0	17.1 ± 18.0	−1.3	0.81	−5.7	0.20	−4.4	0.10	−5.0	0.04
CT at 24 h (*n* = 33)	27.9 ± 52.4	29.5 ± 42.3	28.7 ± 4.2		−1.6	0.80	10.8	0.15	12.4	0.03	11.6	0.05

**Table 2:** Lin’s concordance correlation coefficient for CT on admission and at 24 h. O1, observer 1; O2, observer 2.

		Concordance Correlation	Confidence Interval (CI)	*p*-Value
CT on admission (*n* = 33)	O1 vs. O2	0.490	0.236–0.743	<0.001
	O1 vs. e-ASPECTS	0.576	0.409–0.744	<0.001
	O2 vs. e-ASPECTS	0.756	0.629–0.883	<0.001
	Mean (O1, O2) vs. e-ASPECTS	0.794	0.691–0.898	<0.001
CT at 24 h (*n* = 33)	O1 vs. O2	0.724	0.564–0.884	<0.001
	O1 vs. e-ASPECTS	0.399	0.252–0.546	<0.001
	O2 vs. e-ASPECTS	0.492	0.339–0.645	<0.001
	Mean (O1, O2) vs. e-ASPECTS	0.495	0.350–0.640	<0.001

## Data Availability

Data used in the present study may be available to investigators with appropriate data transfer agreements upon reasonable request to the corresponding author.

## Supplementary Materials

The following supporting information can be downloaded at: https://www.mdpi.com/article/10.3390/brainsci15060560/s1, Supplementary Table S1. Baseline clinical characteristics of patient cohort. Supplementary Table S2. Infarct volume measured from CT on admission and at 24 h in patients with large vessel occlusion (LVO) using the ABC/2 method and e-ASPECTS. Supplementary Table S3. Lin’s concordance correlation coefficient for CTs performed on admission and at 24 h in patients with large vessel occlusion (LVO) using the ABC/2 formula and e-ASPECTS.

## Abbreviations

AAIV = automatically derived acute ischaemic volume; AIS = acute ischaemic stroke; CI = confidence interval; EVT = endovascular thrombectomy; ICH = intracerebral haemorrhage; IV tPA = intravenous tissue plasminogen activator; NCCT = non-contrast computerised tomography (NCCT); MRI = magnetic resonance imaging; DWI-MRI = diffusion-weighted magnetic resonance imaging.
