# Supplementary material for: Comparative Analysis of the ABC/2 Score and e-ASPECTS Software in the Determination of Acute Ischaemic Stroke Volume from Non-Contrast CT
Source: Brain Sci. 2025 May 24;15(6):560. doi: 10.3390/brainsci15060560 (PMC12190421; doi:10.3390/brainsci15060560)
Supplement: Supplementary file 1 [file brainsci-15-00560-s001.zip › brainsci-3604698-supplementary.pdf]

Comparative analysis of the ABC/2 score and e-ASPECTS software in the determination of acute ischaemic stroke volume from non-contrast CT

Supplementary Materials

Supplementary Table S1. Baseline clinical characteristics of patient cohort.

Supplementary Table S2. Infarct volume measured from CT on admission and at 24 h in patients with large vessel occlusion (LVO) using the ABC/2 method and e-ASPECTS.

Supplementary Table S3. Lin's concordance correlation coefficient for CTs performed on admission and at 24 h in patients with large vessel occlusion (LVO) using the ABC/2 formula and e-ASPECTS.

Supplementary Table S1. Baseline clinical characteristics of patient cohort.

**Characteristics**	**Stroke Cohort (*n* = 33)**
**Age (years), mean ± SD**	75 ± 10.8
**Female sex, n, (%)**	14 (42%)
**Barthel index (mean) ± SD**	99 ± 2.2
**NIHSS on admission, mean ± SD**	14 ± (6.3)
**SBP on admission, mean ± SD**	159 ± (21)
**Risk factors**	
Atrial fibrillation, n (%)	8 (24%)
Diabetes Mellitus, n (%)	3 (9%)
Family history of stroke, n (%)	9 (27%)
Hypertension, n (%)	22 (67%)
Dyslipidaemia, n (%)	21 (64%)
Ischaemic heart disease, n (%)	13 (39%)
Active smoker, n (%)	7 (21%)
Alcohol excess, n (%)	4 (12%)
Peripheral arterial disease, n (%)	2 (6%)
**Qualifying event**	
Ischaemic stroke-TOAST classification	
Cardioembolism, n (%)	17 (52%)
Large artery atherosclerosis, n (%)	5 (15%)
Other known aetiology, n (%)	1 (3%)
Unknown aetiology, n (%)	10 (30%)
**Reperfusion therapy**	
Tissue plasminogen activator, n (%)	23 (70%)
Endovascular mechanical thrombectomy, n (%)	23 (70%)
**Occlusion site**	
M1	19 (58%)
M2	8 (24%)
M3	1 (3%)
Tandem	2 (6%)
ICA	2 (6%)

No occlusion	1 (3%)

Supplementary Table S2. Infarct volume measured from CT on admission and at 24 h in patients with large vessel occlusion (LVO) using the ABC/2 method and e-ASPECTS software (ml).

**Large vessel occlusion**	**O1**	**O2**	**Mean (O1, O2)**	**e-ASPECTS**	**O1 vs. O2**		**O1 vs. e-ASPECTS**		**O2 vs. e-ASPECTS**		**Mean(O1, O2) vs. e-ASPECTS**	
	**Volume (ml)**				**Diff (ml)**	***p***	**Diff (ml)**	***p***	**Diff (ml)**	***p***	**Diff (ml)**	***p***
CT on admission (n = 31)	12.2±35.1	13.6±26.3	12.9±26.7	17.8±18.4	-1.3	0.81	-5.6	0.23	-4.2	0.14	-4.9	0.06
CT at 24 h (n = 31)	29.7±53.6	31.4±43.1	30.5±45.1		-1.7	0.80	11.9	0.14	13.5	0.03	12.7	0.04

Supplementary Table S3. Lin's concordance correlation coefficient for CTs performed on admission and at 24 h in patients with large vessel occlusion (LVO) using the ABC/2 formula and e-ASPECTS software.

		Concordance correlation	Confidence Interval (CI)	p-value
**CT on admission** (*n* = 31)	O1 vs. O2	0.484	0.220 - 0.748	<0.001
	O1 vs. e-ASPECTS	0.573	0.400 - 0.746	<0.001
	O2 vs. e-ASPECTS	0.754	0.623 - 0.886	<0.001
	Mean (O1, O2) vs. e-ASPECTS	0.794	0.687 - 0.900	<0.001
**CT at 24 h** (*n* = 31)	O1 vs. O2	0.717	0.549 - 0.885	<0.001
	O1 vs. e-ASPECTS	0.392	0.411 - 0.799	<0.001
	O2 vs. e-ASPECTS	0.483	0.323 - 0.644	<0.001
	Mean (O1, O2) vs. e-ASPECTS	0.487	0.335 - 0.638	<0.001
